# Estimating the Size of Illicit Tobacco Market in Lithuania: Results from the Discarded Pack Collection Method

**DOI:** 10.1093/ntr/ntad013

**Published:** 2023-01-21

**Authors:** Vaida Liutkutė-Gumarov, Lukas Galkus, Laura Miščikienė, Janina Petkevičienė, Mindaugas Štelemėkas, Tadas Telksnys, Justina Vaitkevičiūtė, Hana Ross

**Affiliations:** Faculty of Public Health, Health Research Institute, Lithuanian University of Health Sciences, Kaunas, Lithuania; Faculty of Public Health, Health Research Institute, Lithuanian University of Health Sciences, Kaunas, Lithuania; Faculty of Public Health, Health Research Institute, Lithuanian University of Health Sciences, Kaunas, Lithuania; Department of Health Management, Faculty of Public Health, Lithuanian University of Health Sciences, Kaunas, Lithuania; Faculty of Public Health, Health Research Institute, Lithuanian University of Health Sciences, Kaunas, Lithuania; Department of Preventive Medicine, Faculty of Public Health, Lithuanian University of Health Sciences, Kaunas, Lithuania; Faculty of Public Health, Health Research Institute, Lithuanian University of Health Sciences, Kaunas, Lithuania; Department of Preventive Medicine, Faculty of Public Health, Lithuanian University of Health Sciences, Kaunas, Lithuania; Faculty of Public Health, Health Research Institute, Lithuanian University of Health Sciences, Kaunas, Lithuania; Faculty of Public Health, Health Research Institute, Lithuanian University of Health Sciences, Kaunas, Lithuania; Research Unit of the Economics of Excisable products, University of Cape Town, Cape Town, South Africa

## Abstract

**Introduction:**

For decades in Lithuania, the threat of illicit trade has been used to weaken evidence-based tobacco-control policies and to undermine efforts to reduce smoking prevalence and its attributable burden, while also depriving the government of much-needed tax revenue. The aim of this study is to estimate the size of the illicit cigarette market in Lithuania using data from a nationally representative discarded pack collection.

**Aims and Methods:**

The study employed a two-stage cluster design by first randomly selecting 65 well-defined population settlements (30 cities and 35 townships), representing both urban and rural areas, in all 10 counties in Lithuania. Next, we randomly selected 358 polling districts within these settlements. Each polling district had one route along which discarded packs were collected between September 2019 and 2020.

**Results:**

In total, 28.9% (95% CIs = 27.7 to 30.1) of discarded cigarette packs were classified as illicit. The vast majority (90.1%) of illicit packs originated from Belarus with most (86.9%) packs produced in the Grodno Tobacco Factory Neman. Tax stamps were present on 93.6% of legal packs and also on 76% of illegal packs.

**Conclusions:**

Data from this study suggest that the illicit cigarette trade in Lithuania is more widespread than indicated by other methods and primarily supplied by the neighboring Belarus state-owned tobacco factory in Grodno. This signals the need to adopt Belarus-specific border control and security measures.

**Implications:**

This study presents data from the first national industry-independent study on illicit tobacco trade in Lithuania using discarded cigarette pack collection method. As customs seizure data show, our results also indicate that the illicit cigarette market is primarily supplied by Belarus state-owned Grodno Tobacco Factory Neman known for filling Europe with cheap cigarettes. An estimate derived from this study is higher than both the industry-independent estimate obtained by the survey method and the estimates offered by the tobacco industry. This adds to the evidence that the difference in estimates obtained by different methods reflects the strengths and weaknesses of each. The study also demonstrates the impact of a rogue neighbor on the illicit market in an adjacent country and offers suggestions on how to address it.

## Introduction

The illicit trade in tobacco products is more frequent in European countries with a land or sea border with Ukraine, Russia, Moldova, or Belarus, which are major suppliers of cheap and illicit cigarettes in Europe.^1^ Lithuanian borders with Belarus and the Russian region of Kaliningrad account for one-fifth of the external border of the European Union (EU) in the East, thereby increasing the risk of illicit cigarettes leaking into the EU market.

Local authorities recognize Lithuania as a country of transit, transshipment, temporary storage, and preparation for further transportation of illicit tobacco products to Western Europe,^2^ where cigarettes are more expensive. The weighted average price of the standard cigarette pack in Lithuania was relatively low (€3.57) in 2020 in comparison to other EU countries, such as Ireland (€12.06), France (€8.57), or Finland (€7.71).^3^ Large differences in cigarette prices persist in the EU despite the attempt by the European Council Directive 2011/64/EU to achieve convergence by introducing a minimum excise tax “floor” for tobacco products. The price gaps undermine the efficient functioning of the EU’s internal market as well as the effectiveness of the EU tobacco-tax policy.^4^

Until recently, Klynveld Peat Marwick Goerdeler’s (KPMG) estimates commissioned by Philip Morris International and published in project Stella reports were the only estimates of the extent of the illicit cigarette market in Lithuania. KPMG claims that illicit cigarettes comprised 20.2% of the total cigarette market in 2020, the third-highest illicit market share in the European region.^5^ Another industry-funded report published in 2019 names Latvia and Lithuania as countries with the highest rates of illicit cigarette consumption in the EU, 23% and 19%, respectively.^6^

The tobacco industry (TI) claims that it works actively to solve the problem of illicit trade in Lithuania. It sponsors various media and social campaigns, focusing on illicit trade while building a strong partnership with both the government and the non-governmental sector.^7^ After building the new factory in Klaipėda in 1997, Philip Morris International became one of the largest investors in Lithuania. Therefore, it is not a surprise that Lithuania was the first EU country to adopt *Codentify*, the TI’s tracking and tracing technology, which has shown no evidence of its effectiveness in controlling illicit trade.^8^ In 2015, Philip Morris Baltic donated new SUVs and equipment worth more than half a million euros to the State Border Guard Service,^9^ while in 2016–2019, tobacco manufacturers donated 6.2 million euros to purchase new equipment for the Lithuanian customs, the state border guard service, and the police department. Existing agreements between the European commission and transnational tobacco companies (British American Tobacco and Imperial Tobacco) to cooperate in combating illicit cigarettes schedule annual fixed payments to the EU Member States till 2030,^10^ and the industry’s interference continues through the Philip Morris International *Impact* initiative that focuses on illicit trade and related crimes.^11^ Four recipients from Lithuania have received grants from the Philip Morris International *Impact* since 2016.

The TI extensively uses the threat of illicit cigarette trade to postpone, revoke, or simply weaken evidence-based tobacco-control policies in Lithuania, and tax policies in particular. For example, PM Baltic, JTI Baltic, the National Association of Tobacco Producers, industry-funded entities such as the Lithuanian Free Market Institute and the “Lithuania without a shadow” initiative point out to the Government and the Parliament that there is a causal link between tax increases and illicit trade.^12–14^ This undermines efforts to reduce smoking prevalence and its associated public health and economic burden in Lithuania, while also depriving the Government of much-needed tax revenue. This TI interference motivated the State Public Health Promotion Fund in 2018 to publish a call for industry-independent research on the illicit cigarette market in Lithuania.

In this context, we performed the first industry-independent study on illicit trade in Lithuania to estimate the magnitude of the illicit cigarette market. We used two direct methods—an observation of cigarette packs during a survey of smokers and an observation of empty packs collected from the street—to generate nationally representative estimates. The results of the survey were published in 2020.^15^ This paper examines the proportion of illicit cigarette packs in a sample of discarded cigarette packs.

## Methods

The pack collection was carried out in three rounds: Round 1 (September–November 2019), round 2 (February–March 2020), and round 3 (June–September 2020). Initially, we planned only two rounds, one in autumn and one in spring, but the second round was interrupted by the coronavirus disease 2019 pandemic as a national lockdown was introduced from March 16 to June 16, 2020. Fieldworkers were only able to continue pack collection later in the summer and autumn of 2020.

To determine the nationally representative sample size we used a 95% confidence interval with a margin of error of 1% and assumed that the illicit market share constitutes 20% of the total market based on the KPMG’s project Stella 2019 estimate.^16^ This resulted in a sample size of 6147 packs. We collected 810 packs during the first round, 388 packs during the second, and the majority, 4529 packs, in the third round.

Following the legal definition of cities (more than 3000 citizens) and townships (more than 1000, but less than 3000 citizens), we randomly selected 3 cities and 4 townships in each of the 10 counties in Lithuania representing urban and rural areas, respectively. Since not every county had four townships meeting the definition, we ended up with only 35 townships and 30 cities. Villages were not included in the study because of the lack of public places where littering could occur. Some cities and townships were situated in border regions.

The routes were determined by a random draw of polling districts, as defined by the Central Electoral Commission of the Republic of Lithuania. Each selected polling district represented a single route that was about 3 km long and was planned to avoid bus stops, train stations, and other areas frequented by tourists. The final route was then determined by actual walking conditions, road repair works, and any other factors outside our control. In total, there were 358 routes, 323 in the cities, and 35 in townships tracked using either the *Endmondo* or the *MapMyWalk* applications. All planned and completed routes are archived and available on the Health Research Institute website.^17^

In total, there were 15 fieldworkers instructed to collect all littered packs of tobacco products either found on the ground or in the waste bins using trash picker tools. Discarded packs were placed in separate bags for every route, and labeled with the date, location, route name, and the name of the fieldworker. The collected packs were examined independently by two team members who entered the data into an Excel sheet. The two criteria determined a pack intended for the local market: (1) the presence of a Lithuanian tax stamp with the text “Sold in Lithuania” and ‘UAB Garsu pasaulis’ (a tax stamp in Lithuania is affixed under the cellophane and that cannot be removed; a pack cannot be opened without damaging the tax stamp), and (2) the presence of state required health warning (covers 65% of both the front and the back of a pack; the health warning is in Lithuanian). After the state tax inspectorate confirmed that there are no recorded cases of counterfeit tax stamps in Lithuania, all packs with compliant stamps were considered licit. We assumed that packs without a cap that carries the tax stamp were intended for the local market if the second criterion was met. This was based on the observation that all packs with a cap that had the correct health warning also had the correct tax stamp.

Packs not intended for the local market are referred to as non-domestic cigarette packs. Given the harmonized minimum excise duty of cigarettes in the EU (including the UK, Switzerland, and Norway) and a relatively high legal import limit of 800 cigarettes (40 packs) per person within the EU, the few packs that we found from the EU countries were considered licit inflows. We also found a few packs with duty-free signs and considered them legal. This was based on a smokers’ survey,^15^ in which 1.1% of smokers reported buying cigarettes in duty-free stores or abroad during the last 30 days. In summary, only packs that originated from a non-EU country and did not have any duty-free marking were considered illicit. The origin of a pack was identified by a tax stamp and/or a health warning. If a pack had neither a tax stamp nor health warning, we used other features, such as the quitline phone number, the website for cessation services, the name of the manufacturer, brand, and/or duty-free marking to determine the origin of the pack.

To address the under- and over-collection of packs in ­certain areas, the data were weighted by a coefficient ${\text{ω}}_{\text{i}}\text{=}\frac{{\text{E}}_{\text{i}}}{{\text{O}}_{\text{i}}}$, where $E_{\text{i}}{\text{ and O}}_{\text{i}}$ denotes the expected and collected packs (both illicit and licit) in each city and township, respectively.

Statistical data analysis was performed using SPSS, Version 27, and Microsoft Excel 2019. A statistical signiﬁcance level (*p* values) of .05 was chosen to test the hypotheses. The categorical variables were presented as percentages and compared using a Chi-squared (χ^2^) and a Z-test with Bonferroni correction. The Clopper–Pearson interval was used to calculate binomial 95% confidence intervals.

## Results

In total, the fieldworkers walked at least 1074 kilometers and collected a total of 6517 packs, of which 5727 were cigarette packs and 790 were packs of other tobacco products, including 7% (*n* = 455) *Heets* packs, 4.9% (*n* = 322) cigarillo and cigar packs, and 0.2% (*n* = 13) nicotine salts packs. Among the other tobacco product packs, 97.6% (*n* = 771) were domestic. Only one pack of cigarillos and one pack of *Heets* were non-domestic. The majority (69.2%; *n* = 9) of nicotine salt packs were non-domestic, with the highest proportion (46.2%; *n* = 6) originating in the United Kingdom.

Overall, 69.3% of discarded cigarette packs were intended for the domestic market, 26.1% originated in Belarus, 0.4% were from the EU, Switzerland, the United Kingdom, or Norway, and 1.3% of the packs had duty-free stamps (Table 1). The vast majority (90.1%) of illicit packs originated in Belarus. Seven percent of illicit packs originated in Russia.

**Table 1.** Percent of Discarded Illicit Packs by Country of Origin

Country of origin	Unweighted				Weighted	
	Packs collected (*n*)	Packs collected (% of total)	Illicit packs (*n*)	Illicit packs (% of total)	Packs collected (% of total)	Illicit packs (% of total)
Armenia	1	0.02	1	0.06	0.02	0.05
Belarus	1492	26.05	1492	90.1	27.14	91.1
Belgium	1	0.02	0	0.00	0.02	0.00
Denmark	2	0.03	0	0.00	0.02	0.00
Duty-Free	77	1.34	0	0.00	1.53	0.00
Egypt	2	0.03	2	0.12	0.03	0.11
Georgia	1	0.02	1	0.06	0.02	0.05
Germany	5	0.09	0	0.00	0.05	0.00
Greek	1	0.02	0	0.00	0.02	0.00
Italy	1	0.02	0	0.00	0.02	0.00
Korea	1	0.02	1	0.06	0.02	0.05
Latvia	4	0.07	0	0.00	0.03	0.00
Lithuania	3968	69.29	0	0.00	68.33	0.00
The Netherlands	1	0.02	0	0.00	0.02	0.00
Norway	4	0.07	0	0.00	0.08	0.00
Poland	2	0.03	0	0.00	0.02	0.00
Russia	115	2.01	115	6.94	2.06	6.94
Serbia	12	0.21	12	0.73	0.1	0.33
Switzerland	2	0.03	0	0.00	0.03	0.00
United Kingdom	3	0.05	0	0.00	0.03	0.00
Ukraine	10	0.17	10	0.6	0.1	0.33
Not defined	22	0.39	22	1.33	0.31	1.04
**Total**	**5727**	**100**	**1656**	**100**	**100**	**100**

The origin of packs was primarily determined by the tax stamp and health warning (Table 2) although one-fifth (21.8%) of the packs were assigned an origin using the tax stamp only. Almost all (95.7%) Lithuanian packs had both tax stamps and health warning present. While the majority (81.8%) of Belarusian packs had a tax stamp, one-fifth (17.4%) of them did not have it but had a producer listed on the pack. Tax stamps were present on 93.6% of legal packs, but also on 76% of illegal packs.

**Table 2.** Distribution of Characteristics Used to Define Pack Origin by Country of Origin

Country of origin	Characteristics used to define pack origin							
	Health warnings only (%)	Health warning and producer (%)	Producer only (%)	Producer and brand (%)	Special duty-free marking only (%)	Tax stamp only (%)	Tax stamp and health warning (%)	Total (*n*; [%])
Armenia							100	100 (1)
Belarus		0.1	17.4	0.7		81.8		100 (1492)
Belgium							100	100 (1)
Denmark	50						50	100 (2)
Duty-Free					100			100 (77)
Egypt	50						50	100 (2)
Georgia	100							100 (1)
Germany	40						60	100 (5)
Greek							100	100 (1)
Italy	100							100 (1)
Korea			100					100 (1)
Latvia	50						50	100 (4)
Lithuania	4.3						95.7	100 (3968)
The Netherlands							100	100 (1)
Norway	50	50						100 (4)
Poland	50						50	100 (2)
Russia	2.6	46.1	32.2			17.4	1.7	100 (115)
Serbia			100					100 (12)
Switzerland			50				50	100 (2)
United Kingdom	100							100 (3)
Ukraine						50	30	100 (10)
**Total (%)**	**3.3**	**1.0**	**5.4**	**0.2**	**1.4**	**21.8**	**66.5**	**100 (5705)** ^*^

^*^We were not able to determine the origin of 22 packs.

The unweighted results show that 28.9% (95% CI = 27.7 to 30.1) of the discarded cigarette packs were illicit (Table 3). The townships had a higher share of illicit packs compared to the cities, but the difference was not statistically significant for the unweighted results. Illicit packs were more prevalent in non-border regions than in border regions, but again the difference was not statistically significant for the unweighted results. When comparing the share of illicit cigarette packs among municipalities based on their specific border, the highest proportion of illicit cigarette packs was found in municipalities with a state border with Poland and Belarus, and this holds for both unweighted and weighted results. A higher proportion of illicit packs was found prior to the coronavirus disease 2019 lockdown (introduced on March 16, 2020 and lasting until June 16, 2020), even though this result is not statistically significant when the data are weighted.

**Table 3.** Analysis of Discarded Cigarette Packs

Groups	Unweighted			Weighted		
	Packs collected (*n*)	Illicit packs (%)	95% CI	Packs collected (*n*)	Illicit packs (%)	95% CI
Total	5727	28.9	27.7 to 30.1	6147	29.8	28.7 to 30.9
*Type of residential area*						
Cities	5017	28.8	27.6 to 30.1	4119	28.9	27.5 to 30.3
Townships	710	29.6	26.2 to 33.1	2028	31.7	29.7 to 33.7
Chi-squared (χ^2^) test		*p* = .678			*p* = .022	
*Type of municipality*						
Border	897	27.2	24.3 to 30.2	1178	33.5	30.8 to 32.6
Non-border	4830	29.2	28.0 to 30.5	4969	28.9	27.6 to 30.2
Chi-squared (χ^2^) test		*p* = .218			*p* = .002	
*Border*						
Municipalities with a state border with Latvia	386	25.9^1^	21.6 to 30.6	432	31.9^1^	27.5 to 36.3
Municipalities with a state border with Belarus	200	37.0^2^	30.3 to 44.1	241	43.0^2^	38.3 to 47.7
Municipalities with a state border with Poland	24	41.7^3^	22.1 to 63.4	82	41.5^3^	30.8 to 52.2
Municipalities with a state border with Russia (Kaliningrad)	336	22.0^4^	17.7 to 26.8	348	22.7^4^	18.3 to 27.1
Municipalities with no border	4830	29.2	28.0 to 30.5	4969	28.9	27.6 to 30.2
Chi-squared (χ^2^) test	Comparison of discarded cigarette packs collected in the municipality with state border and packs collected in municipalities with no border; Unweighted ^1^*p* = .166; ^2^*p* = .018; ^3^*p* = .182; ^4^*p* = .005; Weighted ^1^*p* = .185; ^2^*p* < .001; ^3^*p* = .013; ^4^*p* = .013					
*Pack collection round*						
Round 1 (September–November 2019)	810	29.8	26.6 to 33.0	899	28.8	25.8 to 31.8
Round 2 (February–March 2020)	388	38.7*	33.8 to 43.7	194	38.7*	31.8 to 45.6
Round 3 (June–September 2020)	4529	27.9	26.6 to 29.3	5054	29.6	28.3 to 30.9
Z-test with Bonferroni correction	* *p* < .05 significant difference between Round1 and Round2; Round2 and Round3					
coronavirus disease 2019 *lockdown*						
Before the lockdown (September 2019–March 2020)	1198	32.6	30.0 to 35.4	1093	30.6	27.9 to 33.3
After the lockdown (June–September 2020)	4529	27.9	26.6 to 29.3	5054	29.6	28.3 to 30.9
Chi-squared (*χ*^2^) test		*p* = .001			*p* = .550	

CI = confidence interval.

We found 55 different cigarette brands, with Chesterfield, Winston, and Marlboro dominating the market (Table 4). All packs of Lucky Strike, Mark Adams No1, Chesterfield, and LD cigarettes were licit. On the other hand, all packs of LF Light Fight, Minsk, Monus, New Line, NZ Gold, Премьер [Premier], Fest, and Korona were illicit. Cigarette brands produced in Belarus at the Grodno Tobacco Factory Neman (Премьер, Minsk, Fest, and NZ Gold; *n* = 1656) formed the largest proportion (86.9%) of illicit packs. More than half (63%; *n* = 50) of duty-free packs were Winston, which is among the least expensive brands in the airport duty-free shops. These packs were considered legal as explained in the method section. The brand market shares in our sample matched reasonably closely with the brand market shares in Lithuania reported by Euromonitor (Table 5). This increased our confidence in the representativeness of the sample.

**Table 4.** The Share of Illicit Packs by Brand

Brand name	Unweighted		Weighted	
	Packs collected (*n*)	Illicit packs (%)	Packs collected (*n*)	Illicit packs (%)
Camel	99	9.1	95	8.4
LF light fight	59	100	55	100
Lucky strike	23		18	
Mark Adams No 1	35		38	
Marlboro	755	0.3	828	0.2
Minsk	457	100	599	100
Monus	12	100	6	100
New Line	32	100	45	100
NZ Gold	586	100	568	100
Parliament	89	4.5	98	5.1
Премьер [Premier]	20	100	25	100
Chesterfield	776		828	
Rothmans	308	1.6	322	0.9
Winston	755	4.6	799	3.9
Fest	376	100	430	100
Kent	345	1.2	361	1.4
Korona	30	100	26	100
L&M	398	1.0	387	1.0
LD	507		539	
Other ^*^	65	32.3	80	26.3
**Total**	**5727**	**28.9**	**6147**	**29.8**

^*^This category includes cigarette brands with less than 20 packs collected (Allure; American Legend; Best Man; Bond; Break; Cleopatra; Compliment; Credo; Dakota; Davidoff; Esse Change; Floyd; Glamor; Golden Gate; Goldfield; Jin Ling; Karelia; Kastytis; The King Classic; Magic Label; More; Omega; Pall Mall; Paramount; Philip Morris; Prince; Queen; Red & White; Richmond; Saint George; Salem; Slims; Sobranie; Sterling Dual; Style Jade; Vogue; West).

**Table 5.** The 2020 Market Shares of the Top Six Brands Compared to Our Sample

Brand name	Market share, %^*^	Share of licit packs collected during the study, %^**^
Winston	18.0	17.7
Marlboro	17.9	18.5
Chesterfield	17.3	19.1
LD	12.4	12.5
Rothmans	8.8	7.4
L&M	7.7	9.7

^*^Euromonitor International 2021.

^**^The discarded pack collection 2019/20.

## Discussion

Our study used a discarded pack collection method to provide the first industry-independent and transparent estimate of the share of illicit cigarettes in Lithuania. We have found that 28.9% of cigarettes in Lithuania were illicit. Most of them originated in Belarus, in the state-owned Grodno Tobacco Factory Neman factory.

These results are consistent with data from Lithuanian Customs, which reported that cigarettes produced in the Neman factory accounted for 96% of seized contraband cigarettes in 2019, with Queen, Minsk, Fest, and NZ cigarettes being detained most often.^2^ Local authorities recognize Lithuania as a country of transit, transshipment, temporary storage, and preparation for further transportation of illicit tobacco products to Western Europe. Our study suggests that a significant volume of illicit cigarettes circulates in the Lithuanian market.

The proportion of packs classified as illicit in this study is almost 3 times higher than a recent consumer survey estimate of 9.7%.^15^ We attribute the difference to the various strength and weaknesses of the two methods,^18^ because both methods aimed to be nationally representative. The survey results may have been influenced by the fact that only 35% of the sample agreed to show their packs and this reluctance could have been connected to their possession of an illegal pack. Therefore, the survey may have underestimated the scope of tax evasion. On the other hand, the street pack collection captured packs originating from visitors, and therefore possibly overestimated the size of tax evasion. The difference in illicit market share estimates resulting from different methodologies was also observed by other authors,^19–21^ who found that discarded pack survey estimates are higher than survey estimates.

Thanks to the time span of the discarded pack survey, we found that the share of illicit packs was higher before the coronavirus disease 2019 lockdown (32.6%) than after the lockdown (27.9%), even though this result was not statistically significant in the weighted sample. This could be related to the restricted movement of people, especially tourists during the lockdown. For example, the number of tourists from Belarus and Russia, respectively, decreased by 77% and 80% in 2020 compared to the previous year. However, tourists from Belarus and Russia still accounted for 16% of all tourists in 2020.^22^

Even though our observational study has some advantages over the methods based on self-reporting, it is important to acknowledge its limitations. First, this method cannot distinguish between tax avoidance and tax evasion. The availability of cheaper cigarettes in neighboring countries provides an incentive for Lithuanians to shop there.^23–25^ Though the weighted average price per pack in Lithuania, Latvia, and Poland hardly differed in 2020 (€3.57, €3.58, and €3.23, respectively), cigarettes in Russia or Belarus are considerably cheaper. The retail prices for a pack of 20 cigarettes ranged from €0.31 to €1.62^26^ and from €0.07 to €10.40^27^ in Belarus and Russia in 2020, respectively. The 2019 survey found that only 1.1% of smokers in Lithuania bought cigarettes in duty-free shops or abroad,^15^ similar to the situation in other EU countries.^23,24,28^ Even though this estimate is likely to suffer from self-reporting bias, one needs to consider that cross-border shopping in Belarus and Russia is complicated by visa requirements for Lithuanians. This suggests that non-domestic packs are not brought into the country by Lithuanian travelers.

Belarusians, who are on average poorer than Lithuanians, and thus have a lower opportunity cost of their time, are likely to be tempted by the arbitrage opportunity of selling tax-paid Belarusian cigarettes in Lithuania. Belarusian illicit cigarettes were available in Lithuania for €2.20–€2.30 per pack, with the price dropping to €1.70–€1.80 for a bulk purchase in 2020.^29^ The same brands were sold in Belarus legally for around €0.40 to €0.89.^26^ We found that tax stamps were present on 82% of all Belarusian packs. This suggests that these cigarettes were likely legal at some stage in the supply chain. If all smoking visitors to Lithuania from Belarus brought their own cigarettes with them and followed the legal limit of two packs, this would amount to 519 322 packs from Belarus in 2020, or about 0.4% of the legal sales in Lithuania that year. In our 2020 sample, the packs from Belarus constituted 38% of legally sold packs in Lithuania. This suggests that illegal cigarettes are brought to Lithuania from Belarus in an organized fashion. We classified all packs from Belarus as illicit, even though a small fraction of them could have been brought legally by tourists. In that case, we would have slightly overestimated the illicit cigarette market share.

Second, a visual inspection of packs does not allow the detection of counterfeits nor confirm the authenticity of the tax stamps. To the extent that we have missed these features, we have underestimated the illicit cigarette market share. However, we confirmed with the authorities that tax counterfeits in Lithuania are rare and hardly recorded.

The discarded pack collection method is sometimes criticized for focusing primarily on densely populated urban areas. This could potentially lead to the overrepresentation of groups such as tourists or students.^30^ To address this criticism, we expanded our data collection beyond big cities to obtain packs from townships. This presented a challenge since public spaces in less populated settlements are usually very well-kept and do not have a lot of litter. Nevertheless, the proportion of illicit packs we found in townships (29.6%) and cities (28.8%) was very similar. This finding is in fact, consistent with our survey,^15^ where both respondents from towns/villages and big cities expressed similar preferences for illicit cigarettes.

Our estimate of the illicit cigarette market share is higher than the estimate provided by the TI. The 2020 industry-funded KPMG Stella project reports a 22% share of non-domestic products in the empty pack survey and a 20.2% share of the counterfeit and contraband market in Lithuania.^5^ These estimates are supposedly based on 5800 packs collected only in Lithuanian urban cities in September 2020. Based on our experience (we needed to walk 924 kilometers in 26 cities and 26 townships to collect 4529 packs between June and September 2020), such a quantity of empty packs cannot be reasonably obtained in one month unless the packs were also obtained from regular trash collection. The lack of transparency about the methodology used by AC Nielsen Baltics makes it hard to explain how these data were obtained. As we demonstrate in this paper, the methodology can have a profound impact on the estimate. Even though the industry tends to overestimate the illicit market share to advance its arguments against higher tobacco taxes, some industry-independent estimates are higher than industry estimates.^1^ The TI did not know the result of our pack collection study when publishing its 2020 results, and even if it did it would have been difficult to justify such a large increase in illicit trade since the TI reported a 17.7% market share in 2019.

It is becoming increasingly clear that the main reason for the large illicit cigarette market in Lithuania is a state-owned factory in Belarus, Grodno Tobacco Factory Neman, that until recently manufactured cigarettes for British American Tobacco.^31^ British American Tobacco pulled out in 2021 because of the imposition of international sanctions on many entities in Belarus, including Neman.^32^ The U.S. Department of the Treasury,^33^ as well as independent investigative journalists from Lithuania and Belarus,^34^ indicates that the Grodno Tobacco Factory Neman, the Belaruskali enterprise, and Mr. Aliaksey Aleksin, to whom the head of Belarus, Mr. Lukashenko, provided a near monopoly on the production of tobacco products in Belarus, are the key players in supplying illegal cigarettes to Lithuania.^35^ The Lithuanian government has recently terminated its railway contract with the Belaruskali enterprise. It is not clear how much this is going to impact the illicit cigarette market since the most-seized illegal cigarettes from Belarus arrive in cargo trucks.^36^ In addition, the new security measures recently installed at the Belarus–Lithuania border to control the migrant crisis, and thus illegal movement of people across the border, may impact small scale smuggling of illicit goods.^37^ Therefore, it is important to monitor the illicit cigarette trade and its response to these measures. This will generate valuable information not only for Lithuania but for enforcement agencies around the world that deal with a rogue neighbor supplying illicit cigarettes, as we currently see on the borders with Abkhazia^38^ or Paraguay.^39^ If Lithuania could successfully prevent illicit cigarettes from entering the country, the supply channels for illicit cigarettes to the rest of the EU would be limited, since Belarus is the main source of cheap illicit cigarettes in Europe and Lithuania serves as a gateway.

The sizeable market share for illicit cigarettes in Lithuania means that the EU Track & Trace system (T&T) that became operational on May 20, 2019 is not working properly, at least in the Lithuanian context. Despite a 1-year transitional period, 4 months after the full implementation of the EU T&T system we found 32% of packs without the required unique identifiers. It was completely absent in 6% of licit packs and 95% of illicit packs. It is troubling that 35% of the packs (*N* = 7) from other EU countries that we found in Lithuania had no unique identifier.^40^ The EU T&T system failed to ensure that the system is independent of the TI. In Lithuania, for example, the packs’ unique identifier is issued by Atos with Dentsu Aegis as the secondary data provider. Both companies have close ties with TI.^40^ Another concern related to the EU-wide T&T system is its proprietary nature which makes it difficult even for law enforcement authorities to conduct investigations and exchange data. A global T&T as envisioned by the protocol to eliminate illicit trade in tobacco products will never work properly without a mechanism for data exchange.

Despite the T&T system being run by companies with strong TI links, Lithuania could focus on controlling the cigarette supply from Belarus by strengthening the use of intelligence and analytics within the Lithuanian Customs and modernizing border controls by employing surveillance technologies, such as drones and X-ray scanners for cargo vehicles and train carriages. Targeting the distribution of these illegal products and their reexport to the rest of the EU, and putting political pressure on Belarus, should be an integral part of these efforts. The presence of illicit cigarettes in the market is not an obstacle to implementing an evidence-based tobacco-tax policy, as is supported by research findings from other countries.^38,41,42^

## Conclusions

This study indicates that the illicit cigarette trade in Lithuania is larger than previously reported, at 29% of the overall market. Illicit cigarettes are primarily supplied by neighboring Belarus.

In Lithuania, as elsewhere in the world, the TI and its allies use the threat of illicit trade to weaken tobacco-tax reforms and other tobacco-control regulations. Tax policies, the most cost-effective measure to reduce tobacco consumption are often blamed for illicit trade, even though the recent empirical evidence demonstrates no, or at most a very limited, relationship between tax increases and changes in the illicit cigarette market. The presence of illicit cigarettes is not an obstacle to tobacco tax increases that are very much needed in Lithuania, where cigarettes are becoming more affordable.^43^ Higher affordability can stimulate more cigarette consumption with a negative impact on public health and the economy of Lithuania.

## Data Availability

Data used in this study are currently not publicly available but may be shared upon reasonable request to the corresponding author.

## References

[1] Joossens L , Lugo A, La Vecchia C, Gilmore AB, Clancy L, Gallus S. Illicit cigarettes and handrolled tobacco in 18 European countries: a crosssectional survey. Tob Control. 2014;23(e1):e17–e23. 23233420 10.1136/tobaccocontrol-2012-050644 PMC3812425

[2] Lietuvos Respublikos Muitinė. Lietuvos Respublikos Muitinės 2020 *metų veiklos ataskaita* . Retrieved from https://lrmuitine.lt/mport/failai/veikla/veiklos_kryptys/2020_metine_ataskaita.pdf. Accessed November 18, 2021.

[3] European Commission. 2021. *Excise duty tables. Part III Manufactured Tobacco*. Retrieved from https://taxation-customs.ec.europa.eu/system/files/2021-09/excise_duties-part_iii_tobacco_en.pdf. Accessed November 18, 2021.

[4] Evaluation of the Council Directive 2011/64/EU on the structure and rates of excise duty applied to manufactured tobacco. 2020. *European commission.* Retrieved from https://ec.europa.eu/info/law/better-regulation/have-your-say/initiatives/1570-Evaluation-of-the-excise-duties-applied-on-manufactured-tobacco_en. Accessed October 18, 2022.

[5] KPMG. Project Stella. 2020. *Illicit cigarette consumption in the EU, UK, Norway and Switzerland. 2020 Results*. Retrieved from https://www.pmi.com/resources/docs/default-source/stop-illegal-blogs/kpmg-report---illicit-cigarette-consumption-in-the-eu-uk-norway-and-switzerland---2020-results.pdf?sfvrsn=74baf8b7_2. Accessed November 25, 2021.

[6] Aziani A , Dugato M. *ITTP NEXUS in Europe and beyond. Milano: transcrime – università cattolica del sacro cuore*. 2019. Retrieved from https://www.transcrime.it/wp-content/uploads/2019/07/Nexus.Full-report-09072019.pdf. Accessed November 25, 2021.

[7] Liutkute V. Worldwide news and comment. Tob Control. 2016;25(2):125–128. 27336097 10.1136/tobaccocontrol-2016-052984

[8] Hana R , Michael E, Michael Y. Why governments cannot afford codentify to support their track and trace solution. Tob Control. 2018;27(6):706–708. 29367343 10.1136/tobaccocontrol-2017-053970

[9] Lietuvos rytas. *Cigarečių gamintojai pasieniečiams padovanojo visureigių* . Retrieved from https://www.lrytas.lt/verslas/rinkos-pulsas/2015/12/16/news/cigareciu-gamintojai-pasienieciams-padovanojo-visureigiu-2739660. Accessed November 16, 2021.

[10] Joossens L , Gilmore AB, Stoklosa M, Ross H. Assessment of the European Union’s illicit trade agreements with the four major transnational tobacco companies. Tob Control. 2016 May;25(3):254–260. 26022741 10.1136/tobaccocontrol-2014-052218 PMC4853556

[11] Philip Morris International. *PMI IMPACT Report* . 2019. Retrieved from https://www.pmi-impact.com/explore/support/. Accessed November 16, 2021.

[12] Lietuvos Respublikos Seimo Biudžeto ir finansų komitetas. *Pagrindinio komiteto išvada Dėl Lietuvos Respublikos Akcizų įstatymo Nr. IX-569 1, 2, 3, 30 ir 31 straipsnių pakeitimo ir įstatymo papildymo septintuoju skirsniu įstatymo projekto (XIIIP-2213)* . Retrieved from https://e-seimas.lrs.lt/portal/legalAct/lt/TAK/67fb51e0709211e8a76a9c274644efa9?jfwid=sujolkk37. Accessed October 18, 2022.

[13] Nacionalinė tabako gamintojų asociacija. *Dėl Akcizų plano 2022 m. – 2024 m., 2022* . Retrieved from https://lrv.lt/uploads/main/meetings/docs/2113982_imp_82cb6e2d5fc062790626c9af4964c3d5.pdf. Accessed October 18, 2022.

[14] Lietuvos Respublikos Seimo Biudžeto ir finansų komitetas. *Pagrindinio komiteto išvada Dėl Lietuvos Respublikos Akcizų įstatymo Nr. IX-569 1, 2, 3, 30, 31 straipsnių, II ir III skyrių pakeitimo įstatymo Nr. XIII-1327 8 ir 9 straipsnių pakeitimo įstatymo Projekto Nr. XIIIP-4019* . Retrieved from https://e-seimas.lrs.lt/portal/legalAct/lt/TAK/88bb50c009fb11eaa727fba41f42a7e9?jfwid=-cu4zs7oyh. Accessed October 18, 2022].

[15] Liutkute Gumarov V , Galkus L, Petkevicienė J, et al. Illicit tobacco in lithuania: a crosssectional survey. Int J Environ Res Public Health. 2020;17(19):7291. 33036211 10.3390/ijerph17197291 PMC7579345

[16] KPMG. Project Stella. 2019. *Illicit cigarette consumption in the EU, UK, Norway and Switzerland. 2019 Results*. Retrieved from https://www.pmi.com/resources/docs/default-source/stop-illegal-blogs/kpmg-report-illicit-cigarette-consumption-in-the-eu-uk-norway-and-switzerland-2019-results.pdf. Accessed November 25, 2021.

[17] Lietuvos sveikatos mokslų universitetas. Instituto elektroniniai leidiniai. Retrieved from https://archyvas.lsmu.lt/media/dynamic/files/21375/piedas.tuicigareipakelityrimomarrutai.pdf. Accessed ­November 16, 2021.

[18] Ross, H. Understanding and Measuring Tax Avoidance and Evasion: A Methodological Guide. Prepared for the Economics of Tobacco Control Project School of Economics, University of Cape Town and Tobacconomics, Health Policy Center, Institute for Health Research and Policy, University of Illinois at Chicago. Washington DC; 2015.

[19] Stoklosa M , Ross H. Contrasting academic and tobacco industry estimates of illicit cigarette trade: evidence from Warsaw, Poland. Tob Control. 2014;23(e1):e30–e34. 23945214 10.1136/tobaccocontrol-2013-051099

[20] Juarez BS de M , ReynalesShigematsu LM, Stoklosa M, Welding K, Drope J. Measuring the illicit cigarette market in Mexico: a cross validation of two methodologies. Tob Control. 2021;30(2):125–131. 32139405 10.1136/tobaccocontrol-2019-055449 PMC7907567

[21] Szklo AS , Iglesias RM, Stoklosa M, et al. Cross-validation of four different survey methods used to estimate illicit cigarette consumption in Brazil. Tob Control. 2022;31(1):73–80. 33188148 10.1136/tobaccocontrol-2020-056060

[22] Keliauk Lietuvoje. *Turizmo statistika* . Retrieved from https://www.lithuania.travel/lt/news/turizmostatistika. Accessed November 25, 2021.

[23] Stoklosa M. Prices and crossborder cigarette purchases in the EU: evidence from demand modelling. Tob Control. 2020 Jan;29(1):55–60. 30545966 10.1136/tobaccocontrol-2018-054678

[24] Nagelhout GE , van den Putte B, Allwright S, et al. Socioeconomic and country variations in crossborder cigarette purchasing as tobacco tax avoidance strategy. Findings from the ITC Europe Surveys. Tob Control. 2014;23(suppl 101):i30–i38. 23644287 10.1136/tobaccocontrol-2012-050838 PMC4009359

[25] Driezen P , Thompson ME, Fong GT, et al. EURESTPLUS consortium. Crossborder purchasing of cigarettes among smokers in Six Countries of the EURESTPLUS ITC Europe Surveys. Tob Induc Dis. 2018;16(suppl 2):A13. 31516467 10.18332/tid/100411 PMC6661845

[26] Министерство по налогам и сборам Республики Беларусь. *Максимальные розничные цены на сигареты с фильтром* . Retrieved from http://www.nalog.gov.by/ru/tabake/. Accessed November 25, 2021.

[27] федерация налоговая служба. *Сведения о ММРЦ на табачные изделия*. Retrieved from https://service.nalog.ru/tabak.do. Accessed November 25, 2021.

[28] Agaku IT , Blecher E, Filippidis FT, Omaduvie UT, Vozikis A, Vardavas CI. Impact of cigarette price differences across the entire European Union on Crossborder purchase of tobacco products among adult cigarette smokers. Tob Control. 2016;25(3):333–340. 25661415 10.1136/tobaccocontrol-2014-052015

[29] Švedas G. Prevention of illicit trade in tobacco products: experience of lithuania. In: Nowak C. (ed) Combatting Illicit Trade on the EU Border. Springer, Cham.

[30] Gilmore AB , Rowell A, Gallus S, Lugo A, Joossens L, Sims M. Towards a greater understanding of the illicit tobacco trade in Europe: a review of the PMI funded “Project Star” report. Tob Control. 2014;23(e1):e51–e61. 24335339 10.1136/tobaccocontrol-2013-051240 PMC4078702

[31] Open Joint Stock Company Grodno Tobacco Factory Neman. *History of the company* . Retrieved from http://www.tabak.by/en/company/hystory/. Accessed November 25, 2021.

[32] Tobacco Reporter. 2021. *BAT suspends Belarus manufacturing*. Retrieved from https://tobaccoreporter.com/2021/09/15/bat-suspends-manufacturing-in-belarus/. Accessed November 25, 2021.

[33] U.S. Department of The Treasury. *Treasury holds the Belarusian regime to account on anniversary of fraudulent election* . Retrieved from https://home.treasury.gov/news/press-releases/jy0315. Accessed March 26, 2022.

[34] Yarashevich A , Karpeka A, Ratmirova O, Černiauskas Š. *Soon after taking over Belarus’ tobacco industry, oligarch donated luxury cars to Lukashenko regime* . Retrieved from https://www.occrp.org/en/investigations/soon-after-taking-over-belarus-tobacco-industry-oligarch-donated-luxury-cars-to-lukashenko-regime. Accessed March 26, 2022.

[35] Baltic News Service. *Pareigūnai aptiko stambią cigarečių kontrabandą „Belaruskalij“ krovinyje* . Retrieved from https://www.delfi.lt/news/daily/lithuania/pareigunai-aptiko-stambia-cigareciu-kontrabanda-belaruskalij-krovinyje.d?id=87859439. Accessed March 26, 2022.

[36] Gaidamavičius G. Muitininkai pasakė, *ar nebevežant trąšų iš Baltarusijos sustos ir kontrabanda*. Retrieved from https://www.delfi.lt/news/daily/lithuania/muitininkai-pasake-ar-nebevezant-trasu-is-baltarusijos-sustos-ir-kontrabanda.d?id=89451249. Accessed March 26, 2022.

[37] Lietuvos Respublikos vidaus reikalų ministerija. *Pasienyje su Baltarusija pristatytos Lietuvos pastangos siekiant apginti ES išorinę sieną* . Retrieved from https://vrm.lrv.lt/lt/naujienos/pasienyje-su-baltarusija-pristatytos-lietuvos-pastangos-siekiant-apginti-es-isorine-siena. Accessed January 25, 2022.

[38] Little M , Ross H, Bakhturidze G, Kachkachishvili I. Analysis of the illicit tobacco market in Georgia in response to fiscal and non-fiscal tobacco control measures. Tob Control. 2021;0:1–6. 10.1136/tobaccocontrol-2020-056404 PMC9763157 34112646

[39] Iglesias RM , Gomis B, Carrillo Botero N, Shepherd P, Lee K. From transit hub to major supplier of illicit cigarettes to Argentina and Brazil: the changing role of domestic production and transnational tobacco companies in Paraguay between 1960 and 2003. Global Health. 2018;14(1):111. 30454015 10.1186/s12992-018-0413-2 PMC6245621

[40] Liutkute- Gumarov V , Ross H. Tobacco industry compliance with the EU track and tracing system in Lithuania. Tob Control. 2021;0:1–3. 10.1136/tobaccocontrol-2021-056992 34785563

[41] Lavares MP , Ross H, Francisco A, Doytch N. Analysing the trend of illicit tobacco in the Philippines from 1998 to 2018. Tob Control. 2022;31(6):701–706. 33608467 10.1136/tobaccocontrol-2020-056253 PMC9606485

[42] Chisha Z , Janneh ML, Ross H. Consumption of legal and illegal cigarettes in the Gambia. Tob Control. 2020;29(suppl 4):s254–s259. 31147485 10.1136/tobaccocontrol-2019-055055

[43] Statistics Lithuania. *Purchasing power of average monthly net earnings spent on tobacco products* . Retrieved from https://osp.stat.gov.lt/statistiniu-rodikliu-analize#/. Accessed November 25, 2021.

